# Precipitation variation: a key factor regulating plant diversity in semi-arid livestock grazing lands

**DOI:** 10.3389/fpls.2024.1294895

**Published:** 2024-02-28

**Authors:** Yantao Wu, Hao Li, Jiahe Cui, Ying Han, Hangyu Li, Bailing Miao, Yongkang Tang, Zhiyong Li, Jinghui Zhang, Lixin Wang, Cunzhu Liang

**Affiliations:** ^1^ College of Life Sciences, Inner Mongolia University, Hohhot, China; ^2^ Key Laboratory of Ecology and Resource Use of the Mongolian Plateau, Ministry of Education of China, Collaborative Innovation Center for Grassland Ecological Security, School of Ecology and Environment, Inner Mongolia University, Hohhot, Inner Mongolia, China; ^3^ College of Resources Environment and Tourism, Capital Normal University, Beijing, China; ^4^ Inner Mongolia Meteorological Institute, Hohhot, China; ^5^ Alxa League Meteorological Bureau, Bayanhot, China

**Keywords:** grazing intensity, sheep, precipitation concentration index, standardized precipitation evapotranspiration index, species richness, meta-community, grassland ecosystems

## Abstract

Livestock presence impacts plant biodiversity (species richness) in grassland ecosystems, yet extent and direction of grazing impacts on biodiversity vary greatly across inter-annual periods. In this study, an 8-year (2014-2021) grazing gradient experiment with sheep was conducted in a semi-arid grassland to investigate the impact of grazing under different precipitation variability on biodiversity. The results suggest no direct impact of grazing on species richness in semi-arid *Stipa* grassland. However, increased grazing indirectly enhanced species richness by elevating community dominance (increasing the sheltering effect of *Stipa* grass). Importantly, intensified grazing also regulates excessive community biomass resulting from increased inter-annual wetness (SPEI), amplifying the positive influence of annual humidity index on species richness. Lastly, we emphasize that, in water-constrained grassland ecosystems, intra-annual precipitation variability (PCI) was the most crucial factor driving species richness. Therefore, the water-heat synchrony during the growing season may alleviate physiological constraints on plants, significantly enhancing species richness as a result of multifactorial interactions. Our study provides strong evidence for how to regulate grazing intensity to increase biodiversity under future variable climate patterns. We suggest adapting grazing intensity according to local climate variability to achieve grassland biodiversity conservation.

## 1 Introduction

Grasslands provide a variety of habitats for organisms and harbor a large proportion of the world’s plant and animal life forms (Cleland et al., 2013; Petermann and Buzhdygan, 2021; Yan et al., 2023). However, these ecosystems were facing unprecedented threats from climate change and human activities. Herbivorous vertebrates can alter plant biodiversity (species richness) in grassland ecosystems, but the degree and direction of their impact varied greatly depending on grazing intensity and inter-annual variations (Korell et al., 2021). Predicting the impact of herbivores on biodiversity is expected to generate unpredictable effects on ecosystem services and functions (Reich et al., 2012). In an era of rapid species loss caused by global changes and human activities, it is of paramount importance to understand the mechanisms that affect biodiversity (Allan, 2022).

Herbivorous vertebrates play a key role in determining the structure and diversity of grassland plant communities (McNaughton et al., 1989; Olff and Ritchie, 1998; Herrero-Jauregui and Oesterheld, 2018). One theory proposes that ecosystem productivity influences the direction of herbivore effects on plant diversity. For instance, in highly productive systems, herbivores may increase biodiversity by reducing competition for light and promoting seedling establishment (Olff and Ritchie, 1998; Borer et al., 2014; Allan, 2022). In contrast, in low productivity systems, herbivores may decrease species richness by impeding colonization or increasing species extinction (Koerner et al., 2018). Recent studies suggest that changes in community dominance driven by herbivore preference, rather than site productivity, drive grassland biodiversity (Koerner et al., 2018). This theory posits that changes in community dominance alter the competitive abilities among species for above- and below-ground resources in the ecosystem (Olff and Ritchie, 1998). It is noteworthy that grazing intensity has varied direct or indirect effects on diversity. Changes in grazing intensity can also induce shifts in interspecific relationships within the community, thereby influencing biodiversity. Therefore, investigating the role of the stress gradient hypothesis (SGH) in the impact of grazing intensity on biodiversity is essential. The stress gradient hypothesis posits that the net outcome of plant community interactions shifts from negative (competition) to positive (facilitation) as stress increases (Bossuyt et al., 2005; Smith et al., 2009; Adams et al., 2022). Previous studies on the impacts of herbivores on grassland biodiversity have yielded mixed results, with some reporting positive effects, while others report neutral or negative effects (Stohlgren et al., 1999; Koerner et al., 2014; Eldridge et al., 2016; Gao and Carmel, 2020). Therefore, the impact of herbivores on grassland plant diversity is not determined by a single theory but is instead the result of multiple mechanisms acting in concert. The identification of multiple mechanisms driving the impact of herbivores on biodiversity is of significant importance.

A central question in ecology is which biotic and abiotic factors regulate species diversity in plant communities over space and time (Tilman, 2000; Ulrich Sommer and Worm, 2002; Worm et al., 2002). In semi-arid ecosystems, water, especially effective precipitation, is recognized as the primary limiting factor and key driving force for plant biodiversity and productivity (Fu et al., 2017; Shi et al., 2018; Li et al., 2021). These communities face challenging physiological limitations due to water scarcity, making plant productivity more sensitive to precipitation (Korell et al., 2021). The positive correlation between species richness and precipitation has also been confirmed globally, as increased resource/energy availability allows for greater species coexistence (Currie et al., 2004). With rising greenhouse gas levels in the atmosphere causing a warming trend, significant changes in precipitation amount and distribution patterns were expected in arid regions over the coming decades (Cleland et al., 2013; Miao et al., 2020). Interannual precipitation variability and frequency of drought events are widely believed to had increased and are projected to continue increasing in many regions (Durack et al., 2012). Therefore, we need to determine whether the plant diversity across regional precipitation gradients also applies to the temporal changes in biodiversity of a grassland ecosystem driven by interannual variation in precipitation. Additionally, we should pay more attention to the magnitude of changes in the distribution of precipitation within a year (i.e., precipitation concentration index or PCI), as plant available water is not only influenced by precipitation amount but also by the concentration of precipitation events (Knapp et al., 2002; Huxman et al., 2004). Intra-annual precipitation variability (or PCI) may cause a mismatch between water availability and the developmental needs of plants in different growth stages (Voigt et al., 2003; Suttle et al., 2007). For instance, a lack of rainfall during the growing season when plants require more water can result in an inadequate water supply (Wang et al., 2020). Non-concentrated precipitation can prevent species from establishing in a community under high evapotranspiration conditions, thereby affecting species diversity. Additionally, biodiversity often exhibits a certain lag in response to disturbance (grazing intensity) (Trindade et al., 2020). Investigating long-term changes in community structure in response to disturbance will contribute to a better understanding of how grazing influences plant diversity. Therefore, it is necessary to conduct multi-year grazing gradient control experiments to determine how the impact of grazing intensity on biodiversity varies with changes in intra- and inter-annual climate variability.

In this study, we had conducted a long-term multi-gradient grazing experiment to quantify the effects of grazing intensity and climate variability on species richness and to evaluate whether these effects had been mediated by changes in plant community productivity and dominance. Through the time scale of “meta-community”, we had considered all species that appeared in communities under different ecological niches at different times of the year. We expect grazing to influence species richness by altering community productivity and dominance, while interannual climate variability and associated changes in effective moisture supply will enhance or dampen the effects of grazing. We had aimed to address the following two specific scientific questions: (1) How does grazing affect plant diversity? (2) Is the alteration in species richness the combined outcome of grazing intensity, climatic variability, and community structure?

## 2 Methods and data

### 2.1 Study sites

**Figure 1:**
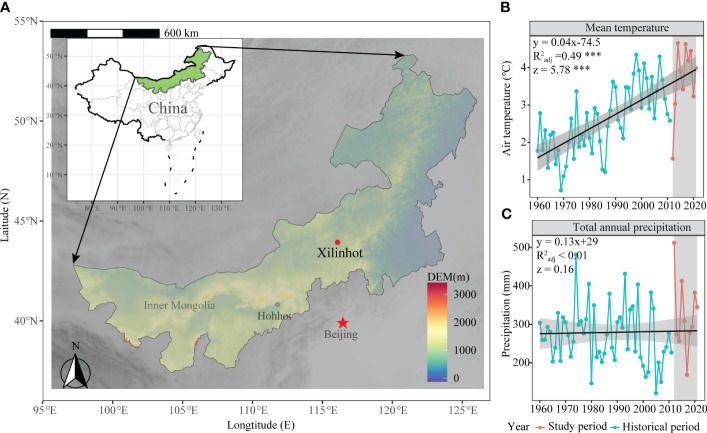
The geographic and historical climatic distributions of grazing manipulation experiments. **(A)** illustrates the distribution of the sample sites on a map. The mean annual temperature in the study area from 1960-2021 is represented in **(B)**, while **(C)** shows the mean annual precipitation during the same period in the study area. '***' represents extremely significant.

**Figure 2:**
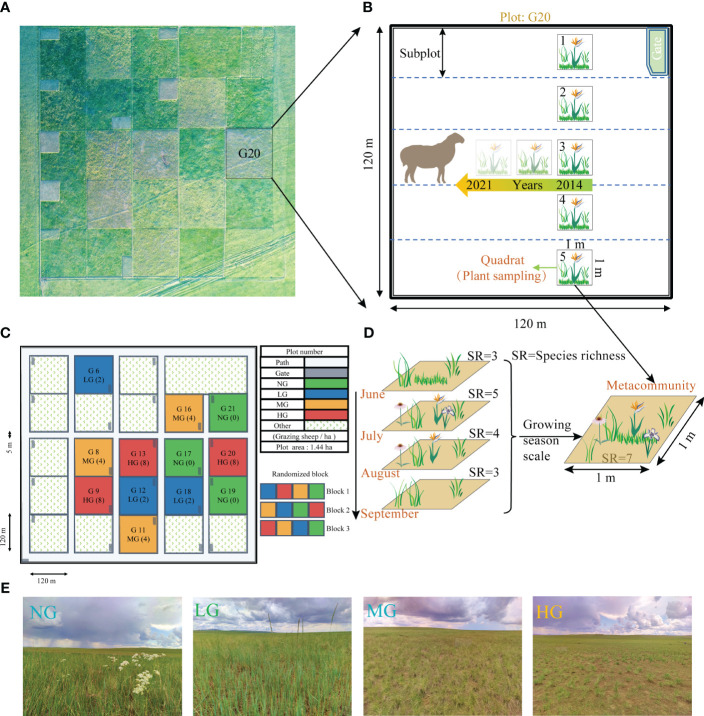
The experimental design for grazing manipulation, long-term plant community survey, and landscape map. **(A)** presents an aerial view of the grazing experiment site, while **(B)** illustrates the long-term monitoring plan for plant community. **(C)** shows the randomized block grazing experiment designed with four treatments, namely low grazing (LG: 2 sheep·ha-1·day-1), medium grazing (MG: 4 sheep·ha-1·day-1), high grazing (HG: 8 sheep·ha-1·day-1), and no grazing (NG: 0 sheep·ha-1·day-1). Each plot covered an area of 1.44 ha and the experiment was conducted over a period of eight years with three replicates per treatment. **(D)** displays an aggregate community at the growing season scale, demonstrating aggregation of four quadrats from the same site in different months. **(E)** shows the landscape map of the four different grazing treatments.

The grazing manipulation experiment had been conducted in Xilinhot City (44°08′N, 116°19′E, 1118m a.s.l), Inner Mongolia, China (**Figure 1A**). From 1960 to 2021, the mean annual temperature (MAT) of this area had increased at a rate of 0.04°C per year, accumulating a total increase of 2.4°C during this period (**Figure 1B**). The mean annual precipitation (MAP) over the past 60 years was approximately 279.54 mm, with nearly 85% occurring during the growth season from May to September (**Figure 1C**; **Figure 2E**). During the study period from 2014 to 2021, this area had experienced a transition from a humid year to a severe drought year and then back to a humid year (**Figure S1**). The biotic community of this area is characterized by a typical grassland dominated by Stipa grandis P. Smirn (a perennial bunchgrass) and Leymus chinensis Trin. Tzvel (a perennial rhizomatous grass). The grazing history of this grassland area had involved low-intensity sheep grazing (approximately 0.5 sheep-day per hectare).

### 2.2 Grazing experiment design

In 2013, we had fenced 12 paddocks of equal-size (120×120 m) and had implemented four grazing intensity treatments from 2014 to 2021: no grazing (NG: 0 sheep·ha−1·day−1), low grazing (LG: 2 sheep·ha−1·day−1), medium grazing (MG: 4 sheep·ha−1·day−1), and high grazing (HG: 8 sheep·ha−1·day−1) (**Figure 2A**). Each grazing treatment had three replicates. Four bouts of grazing were conducted each year during the growing season from June to September using Inner Mongolian Ujimqin sheep (Wu et al., 2022). Each bout lasted for 21 days and followed a particular treatment. Grazing started at 7:00 am and ended at 6:00 pm every day, during which time the sheep had free access to water and minerals (**Figure 2C**).

### 2.3 Data sampling and calculation

#### 2.3.1 Plant sampling

We had virtually divided each paddock (120×120 m) into five subplots (24×120 m). In each subplot, we had randomly placed a 1-m2 quadrat 30 meters from the fence (**Figure 2B**). For each subplot, we had used scissors to clip aboveground living portions of each species from each quadrat and collected them as plant community samples. We had oven-dried these samples at 65°C for 48 hours and weighed them with a 0.01g precision electronic balance to determine the shoot biomass for each species. We collected plant sample data using this method during June, July, August, and September from 2014 to 2021. The focus of this study was the seasonal-scale meta-community, which represented the collection of all species that had appeared within a unit area during the four-month growing season (**Figure 2D**). We had extracted the highest value of shoot biomass recorded for each species during the four months as the biomass of each species in the assemblage community.

#### 2.3.2 Plant diversity

At the local scale (i.e., subplot), we had calculated plant community species richness (q=0), exponentiated Shannon diversity (q=1), and Simpson diversity (q=2) for the seasonal-scale meta-community using three Hill numbers. The number of species in the meta-community for each subplot was calculated as the plant community species richness. The Shannon-Wiener diversity for the community was calculated as $H\text{=-}\sum_{i=1}^{S}p_{i}\times \text{ln}p_{i}$ , where *S* represents the number of species in the meta-community and *p_i_* represents the relative biomass of species *i* in the meta-community. The effective species (i.e., common species) of the community was calculated as the exponentiated Shannon-Wiener of the meta-community. The effective species (referred to as the Shannon diversity hereafter) was calculated as *H’ = e^H^*, where *H* is the Shannon-Wiener diversity of the meta-community. Due to the tendency of dominant species in a community to influence species diversity through resource monopolization, we had quantified community dominance through two methods. The Berger-Parker dominance diversity is the relative biomass of the most abundant species in each subplot’s meta-community (Koerner et al., 2018). Simpson’s dominance index is calculated as $Simp=\sum {\;}_{i}^{S}p_{i}^{2}$ , where *S* represents the number of species in the meta-community and *p_i_* represents the relative biomass of species i in the meta-community.

#### 2.3.3 Climate variables

In our experiment, standardized precipitation evapotranspiration index (SPEI) and precipitation concentration index (PCI) standardized the wetness and precipitation distribution patterns within the year, respectively. We calculated the precipitation concentration index (PCI) to measure the distribution pattern of rainfall during the year (Sloat et al., 2018). The larger the PCI value, the more concentrated the distribution of rainfall during the year, and vice versa. The PCI was calculated as $PCI=\sum_{i}^{m}P_{i}^{2}/{\left(\sum_{i}^{m}P_{i}\right)}^{2}\times 100\%$ , where *P_i_* represents the precipitation in month *i* of the year and m = 12. The peak temperature in the region occurs in July, and whether rainfall is concentrated in July largely determines the growth status of the plant community. Additionally, we calculated the standardized precipitation evapotranspiration index (SPEI) to measure each year’s degree of wetness using the SPEI package in R.

#### 2.3.4 Statistical analyses

To assess the effects of grazing and year on plant diversity and branch biomass, we conducted a repeated-measurements analysis of variance (ANOVA) using the *ez* package in R. In this model, grazing intensity was treated as the between-subjects factor and year as the within-subjects factor. Tukey’s range method was used to test differences in grazing intensity across each year. Additionally, to evaluate the response of species richness to climate and plant community attributes under different grazing intensities, we established ordinary least squares linear regression models (OLS-LM) between climate and community variables (i.e., independent variables) and species richness (i.e., dependent variable).

We also constructed a structural equation model (SEM) to explore how grazing and climate drive species richness through their mediation of plant community dominance and productivity. Shipley’s d-separation test was used to ensure that no significant paths were omitted (p>0.05), and the final SEM model was selected based on the lowest Akaike information criterion (AIC). The SEM was constructed using the *piecewiseSEM* package in R (Lefcheck, 2016). Finally, we used a multiple regression model to evaluate the most important driving factors influencing species richness. To assess the relative importance of each explanatory variable (GI, SPEI, PCI, Berger-Parker, and Shoot biomass) in the best model on species richness, we used the averaged ranking method by running the *relaimpo* package in R to decompose R2. Additionally, we conducted partial correlation analysis to further identify the primary driving factors of species richness and the interdependency among the explanatory variables.

## 3 Results

### 3.1 The impacts of grazing on plant community attributes

**Figure 3:**
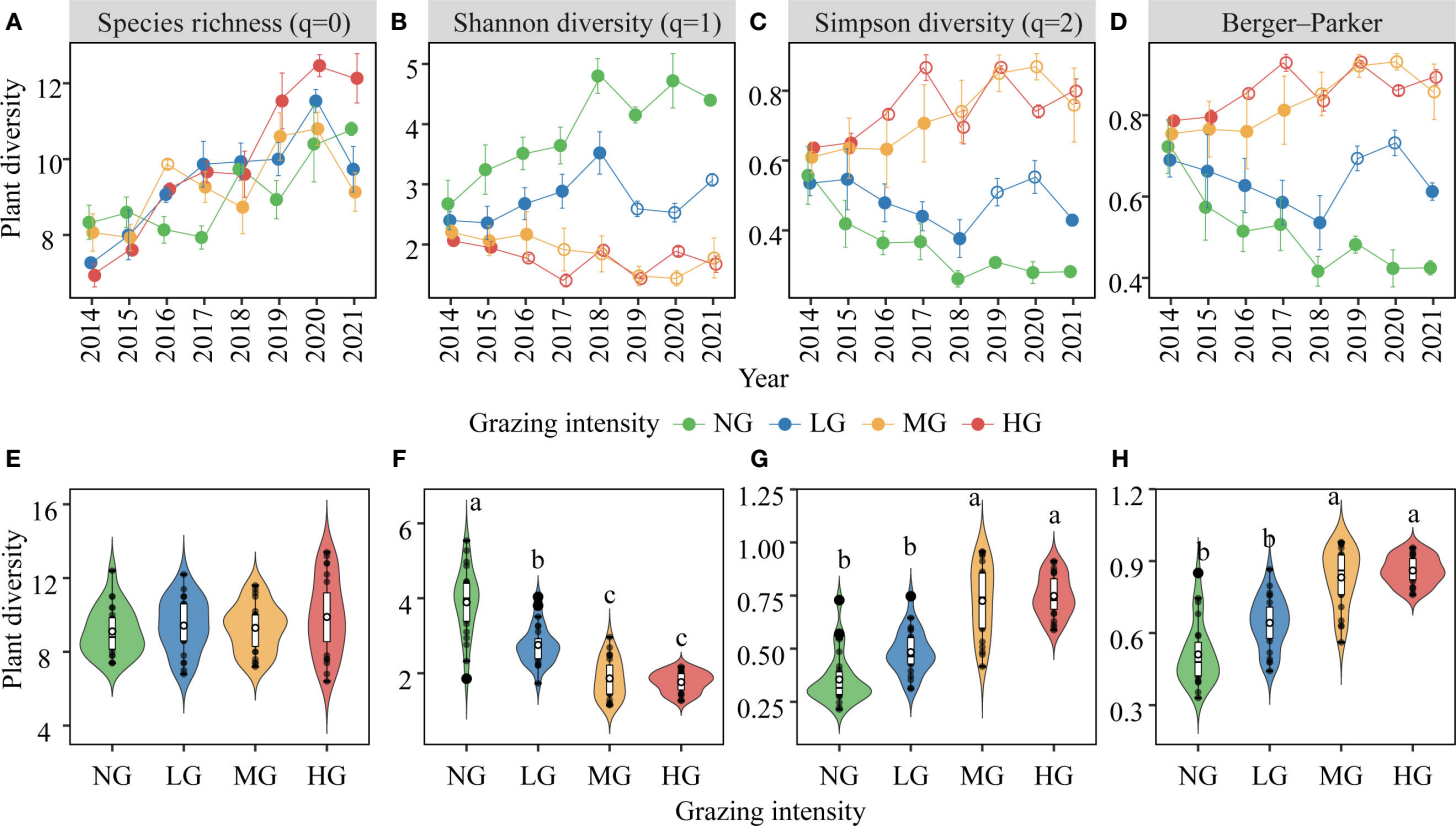
Variations in plant diversity and its response to different grazing intensities over multiple years (mean ± SE, n=3). Shown are the dynamics of species richness **(A, E)**, Shannon diversity **(B, F)**, Simpson diversity **(C, G)**, Berger-parker **(D, H)** with statistics (i.e. Tukey’s range test) indicating the results from the ANOVA models of grazing intensity, year and their interactions (see **Table 1**). Tukey’s range test was used to examine differences between grazing intensities, with unfilled dots indicating significant grazing effects compared to the no-grazing treatment (p< 0.05). Key; NG, no grazing (green); LG, low grazing intensity (blue); MG, medium grazing intensity (yellow); HG, high grazing intensity (red). Different lowercase letters indicate significant differences in plant diversity between pairwise grazing intensities.

Over the years of grazing, there was a significant increase in species richness in the plant community (**Table 1**; **Figure 3A**). However, increased grazing intensity did not have a significant impact on species richness (**Figure 3E**). Furthermore, the interactive effect of the year and the grazing intensity on species richness was also significant (**Table 1**), primarily manifested as follows: the impact of increased grazing intensity on species richness diminished during dry years (e.g., 2017), while it intensified during wet years (2020-2021) (**Table S1**). Additionally, our study indicated that Shannon-Wiener diversity was not only significantly influenced by grazing intensity and year but also by their interaction (**Table 1**). Specifically, compared to no grazing, increased grazing intensity significantly decreased Shannon-Wiener diversity, with this effect becoming more pronounced over the years (**Figures 3B, F**). Our analysis also revealed that plant community dominance, as indicated by Simpson diversity and the Berger-Parker index, was primarily influenced by grazing intensity and its interaction with year (**Table 1**). We found that grazing increased dominance while the no grazing decreased it, with this difference becoming more pronounced over time (**Figures 3C, D, G, H**). Shoot biomass of the plant community was significantly affected only by grazing intensity (**Table 1**), with medium and high grazing being significantly lower than no grazing and low grazing (**Figure S2**).

**Table 1:** Repeated-measures ANOVA results for plant diversity and shoot biomass with grazing intensity (GI) as a between-subjects factor and year (Y) as a within-subjects factor.

	GI		Y		GI*Y	
	F	P	F	P	F	P
Species richness	1.53	0.28	**111.91**	**<0.001**	**6.66**	**<0.001**
Shannon diversity	**30.49**	**<0.001**	**6.28**	**<0.05**	**16.96**	**<0.001**
Simpson diversity	**15.01**	**<0.01**	0.73	0.40	**15.30**	**<0.001**
Berger-Parker	**16.72**	**<0.001**	0.03	0.87	**13.67**	**<0.001**
Shoot biomass	**17.82**	**<0.001**	0.39	0.53	0.35	0.79

F- and P-values were used to represent ANOVA results and statistical significance, respectively. Significant differences (p< 0.05, 95% confidence level, n = 3) are indicated in bold, with GI*Y representing grazing and year interactions.

### 3.2 The combination of grazing and climate factors impels plant diversity

**Figure 4:**
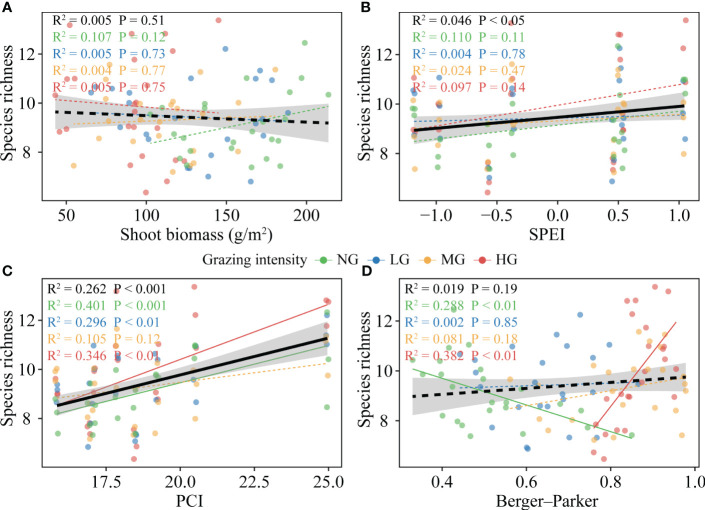
Plant species richness response to biotic and abiotic variables under different grazing intensities. **(A)**, Relationship between shoot biomass and the plant species richness. **(B)**, Relationship between standardized precipitation evapotranspiration index (SPEI) and the plant species richness. **(C)**, Relationship between precipitation concentration index (PCI) and the plant species richness. **(D)**, Relationship between dominance (Berger-Parker dominance index) and the plant species richness. The black line represents the overall relationship from linear models, and the colored line represents the relationship for each grazing intensity (shaded areas indicate 95% confidence intervals). Solid and dashed lines denote significant and insignificant, respectively. Key; NG, no grazing (green); LG, low grazing intensity (blue); MG, medium grazing intensity (yellow); HG, high grazing intensity (red).

**Figure 5:**
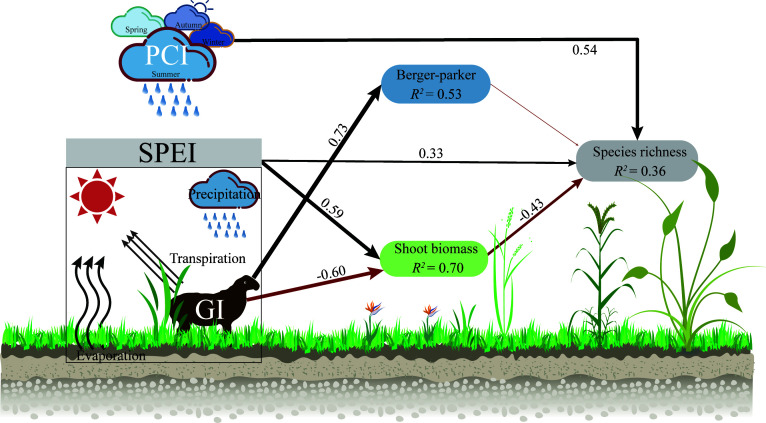
The piecewise structural equation model (pSEM) presents a comprehensive understanding of the impacts of grazing intensity (GI), climate (PCI and SPEI), and community attributes (Berger-parker and Shoot biomass) on species richness, both directly and indirectly. Negative and positive relationships are indicated by the red and black arrows, respectively, with significant associations at p< 0.05. The statistical metrics report Fisher’s C as 10.26, p-value as 0.74, df as 14, and AIC as 38.26. The precipitation concentration index is denoted by PCI, while the standardized precipitation evapotranspiration index is known as SPEI. Grazing intensity is referred to as GI in the model.

The continuous increase in mean annual temperature (MAT) was not conducive to an increase in species richness, with this effect being non-significant across various grazing intensities (**Figure S3B**). We did not observe a significant promoting effect of mean annual precipitation (MAP) on species richness (**Figure S3C**). However, when considering the intra-annual distribution pattern of precipitation, we found that the precipitation concentration index (PCI) had a significant positive effect on species richness, with this effect being more pronounced at higher grazing intensities (**Figure 4C**; **Figure 5**). Additionally, the standardized precipitation evapotranspiration index (SPEI) positively influenced species richness (**Figure 4B**). However, the promoting effect of SPEI on species richness was attenuated by an increase in shoot biomass (**Figure 5**; **Figure S4**).

The increasing grazing intensity had no direct effect on species richness, but indirectly increased it by reducing shoot biomass (**Figure 5**; **Figure S3A**). Shoot biomass had no significant effect on species richness, but the relationship shifted to a significant negative correlation when SPEI was not considered (**Figure 4A**; **Figure 5**; **Figure S4**). The dominance of the community had no clear relationship with species richness, but it should be noted that this relationship was significantly negatively correlated with no grazing, while it turned into a significant positive correlation at high grazing intensities (**Figure 4D**). Conversely, in the absence of grazing, the effective species (Shannon diversity) of the community had a positive promoting effect on species richness (**Figure S3D**).

Plant community species richness was primarily driven by PCI, accounting for 70% of the observed variation. SPEI was the second most influential factor, explaining 14% of the total variance. Branch biomass also plays a significant role in affecting species richness, contributing to 10% of the total variation (**Figure S5**).

## 4 Discussion

Compared to the well-defined spatial distribution pattern of plant diversity (species richness), it is important to determine whether the same driving mechanisms apply on a temporal scale. Our research had shown that in ecosystems with distinct seasonal patterns, precipitation patterns (particularly precipitation concentration index: PCI) within a year have a stronger impact on plant species richness than grazing intensity. Increased grazing intensity did not exert a direct driving effect on species richness. Instead, the regulation of species richness was primarily influenced by changes in aboveground biomass, which were modulated by variations in both grazing intensity and precipitation. Therefore, species richness cannot be determined by a single driving mechanism alone; rather, it is the result of multiple interacting factors (**Figure 5**).

### 4.1 Grazing affect plant diversity

The primary reason for the absence of a decrease in plant species richness due to grazing intensity is the shelter effect generated by the increased dominance of S. grandis. S. grandis is a tall, dense grass species in our ecosystem, which is unpalatable and has defense traits such as long needle-shaped seeds (Liang et al., 2021). Sheep preferentially forage palatable and nutritious species, which indirectly increases the dominance of the community by reducing competition from other species (increasing the dominance of S. grandis) (Liang et al., 2019). Some studies suggest that unpalatable species act as biological refuges by protecting neighboring plants from being eaten (Smith et al., 2009; Kelemen et al., 2019). Our research confirmed this, as high grazing intensity significantly promoted an increase in species richness by increasing the dominance of the community. Under conditions of high grazing intensity, the sheltering effect provided by S. grandis prevents vulnerable species from local extinction due to foraging pressures. Additionally, the relaxation of niche constraints resulting from reduced interspecific competition creates favorable conditions for colonization by new species (Knapp et al., 1999; Bossuyt et al., 2005; van Wieren et al., 2008). However, we found that after grazing was excluded, the community dominance decreased and species richness increased. This inconsistent relationship is due to the shift from mutualistic relationships between species under grazing to competitive relationships when grazing is excluded (Bossuyt et al., 2005; Smit et al., 2009). The stress gradient hypothesis suggests that as environmental stress increases, the importance of positive interactions between species also increases (Gibson, 2009; Smit et al., 2009). Therefore, the theory that livestock, by altering community dominance, influences species richness is not universally applicable across all ecosystems (Koerner et al., 2018). Species interactions are also an important factor that needs to be considered.

### 4.2 Synergistic effects: grazing intensity, climate change, and community structure on biodiversity

The magnitude of community productivity is equally an important factor driving species richness, with productivity primarily regulated by grazing intensity and inter-annual variations in wetness. In arid ecosystems, increased moisture availability, as indicated by higher SPEI (standardized precipitation evapotranspiration index) values, typically leads to greater species richness (Currie et al., 2004). Our research confirms this, as an increase in SPEI may directly provide more ecological niche opportunities for species coexistence (Smith et al., 2022). However, an increase in SPEI also increases the shoot biomass of the community. Generally, under high productivity conditions, taller and fast-growing plants reduce diversity by monopolizing light and shading smaller plants (Borer et al., 2014; Allan, 2022). In our low productivity ecosystem, an increase in shoot biomass is disadvantageous for species richness when not considering the positive effect of SPEI (**Figure S4**). This is because competition among species increases with annual climate moisture (Smith et al., 2022). The presence of livestock mitigated the indirect negative impact of SPEI on species richness caused by an increase in the biomass of branches. (**Figure 5**). One explanation for the importance of productivity is that when herbivores alleviate plant competition, exclusion, and limitations on species establishment, they may increase plant diversity in grasslands (Grubb, 1977; Eskelinen and Virtanen, 2005; Bakker et al., 2006). Therefore, adapting grazing intensity based on climate moisture may be an effective strategy for increasing biodiversity in semiarid grassland ecosystems.

The positive effect of Precipitation Concentration Index (PCI) on plant community species richness is enhanced by an increase in grazing intensity and is the strongest driving factor. Our ecosystem exhibits pronounced seasonality, with over 90% of annual rainfall occurring during the growing season and high evapotranspiration (Liang et al., 2021). Consequently, PCI may be crucial for grassland biodiversity since water availability is influenced not only by precipitation amount but also by precipitation event concentration (Knapp et al., 2002; Sloat et al., 2018). Our study demonstrates that a higher concentration of precipitation events promotes an increase in species richness. This is primarily due to rain and heat coinciding, such as the highest monthly precipitation during 2020-2021 occurring in July, the hottest month (peak growing season). However, we did not find a significant effect of mean annual precipitation on species richness. This is because monthly precipitation during the growing season does not correspond to actual temperature (or evapotranspiration); for instance, in 2015, a large amount of precipitation occurred in June (early growing season) when temperatures were not high. Different water-heat periods throughout the year may result in a mismatch between water availability and plant development requirements at different growth stages (Voigt et al., 2003; Suttle et al., 2007; Wang et al., 2020). Additionally, grazing intensity increases ecological niche space by reducing community coverage. When climate conditions (water-heat coinciding) in a year provide an “opportunity window”, more species (annual plants or perennial seedlings) may establish and grow in the community (Balke et al., 2014; Mortensen et al., 2018). These results provide the first evidence that the distribution pattern of precipitation throughout the year determines the effect of livestock grazing on species richness.

We conclude our discussion with two caveats. Firstly, we failed to conduct *in situ* surveys of community species richness and identity information for each month, which may have led to an overestimation of the temporal-scale meta-community responses to grazing and climate. Secondly, our focus was solely on the impact of sheep grazing on species diversity. However, the impact of different livestock with varying preferences on species diversity can vary greatly (Albon et al., 2007; Tóth et al., 2018). Therefore, we suggest studying a broader range of livestock to investigate how they regulate species diversity at different time scales. Lastly, considering the pronounced seasonality of our ecosystem, the driving effect of precipitation concentration (PCI) on species richness may be limited. In the context of increasingly frequent future extreme weather events (Zhou et al., 2023), it is imperative to investigate the relative driving roles of intra-annual and inter-annual precipitation variability on species richness across various ecosystems.

## 5 Conclusion

The global driving mechanisms of plant diversity in grazing lands are well understood. However, to determine if these spatial patterns apply at the temporal (annual) scale, a thorough understanding of annual variation and its influence by grazing intensity and climate change is necessary. In this study, we focused on the intra-annual precipitation variation patterns, revealing the importance of water-thermal synchronization on plant diversity and elucidating the synergistic effect of grazing intensity on such impacts. Specifically, species richness increases with rising effective precipitation (PCI) and is further enhanced with increasing grazing intensity. Additionally, intra-annual wetness levels (SPEI) improved species richness but also increased community shoot biomass, indirectly weakening this promotional effect. The grazing activity of livestock, however, can alleviate the indirect negative impact of increased humidity on species richness. Thus, we emphasize that the absence of herbivores in semiarid areas can also be detrimental to biodiversity enhancement. In ecosystems with pronounced seasonality, the impact of intra-annual precipitation variation on plant diversity is much greater than that of annual mean precipitation. Future research should focus more on the impact of precipitation variation on grassland ecosystems to better understand driving mechanisms under different climate scenarios.

## Data availability statement

The original contributions presented in the study are included in the article/**Supplementary Material**. Further inquiries can be directed to the corresponding author.

## Ethics statement

The requirement of ethical approval was waived by the Bioethcs Committee of Inner Mongolia University for the studies involving animals because the sheep were sourced through and managed by local herders. The studies were conducted in accordance with the local legislation and institutional requirements.

## Author contributions

YW: Conceptualization, Formal analysis, Investigation, Validation, Visualization, Writing – original draft, Writing – review & editing. HL: Investigation, Writing – review & editing. JC: Data curation, Investigation, Writing – original draft. YH: Conceptualization, Formal analysis, Investigation, Writing – original draft. HL: Investigation, Methodology, Writing – original draft. BM: Conceptualization, Software, Writing – original draft. YT: Investigation, Validation, Writing – original draft. ZL: Conceptualization, Investigation, Methodology, Resources, Validation, Writing – original draft. JZ: Data curation, Investigation, Methodology, Writing – original draft. LW: Formal analysis, Supervision, Validation, Writing – review & editing. CL: Data curation, Formal analysis, Funding acquisition, Methodology, Project administration, Resources, Writing – original draft.

## References

[B1] Adams A. E. Besozzi E. M. Shahrokhi G. Patten M. A. (2022). A case for associational resistance: Apparent support for the stress gradient hypothesis varies with study system. Ecol. letters. 25, 202–217. doi: 10.1111/ele.13917 34775662

[B2] Albon S. D. Brewer M. J. O'Brien S. Nolan A. J. Cope D. (2007). Quantifying the grazing impacts associated with different herbivores on rangelands. J. Appl. Ecology. 44, 1176–1187. doi: 10.1111/j.1365-2664.2007.01318.x

[B3] Allan E. (2022). Shedding light on declines in diversity of grassland plants. Nature 611, 240–241. doi: 10.1038/d41586-022-03458-1 36323896

[B4] Bakker E. S. Ritchie M. E. Olff H. Milchunas D. G. Knops J. M. (2006). Herbivore impact on grassland plant diversity depends on habitat productivity and herbivore size. Ecol. Lett. 9, 780–788. doi: 10.1111/j.1461-0248.2006.00925.x 16796567

[B5] Balke T. Herman P. M. J. Bouma T. J. (2014). Critical transitions in disturbance-driven ecosystems: Identifying windows of opportunity for recovery. J. Ecology. 102, 700–708. doi: 10.1111/1365-2745.12241

[B6] Borer E. T. Seabloom E. W. Gruner D. S. Harpole W. S. Hillebrand H. Lind E. M.. (2014). Herbivores and nutrients control grassland plant diversity via light limitation. Nature 508, 517–520. doi: 10.1038/nature13144 24670649

[B7] Bossuyt B. De FrÉ B. Hoffmann M. (2005). Abundance and flowering success patterns in a short-term grazed grassland: Early evidence of facilitation. J. Ecology. 93, 1104–1114. doi: 10.1111/j.1365-2745.2005.01059.x

[B8] Cleland E. E. Collins S. L. Dickson T. L. Farrer E. C. Gross K. L. Gherardi L. A.. (2013). Sensitivity of grassland plant community composition to spatial vs. Temporal variation in precipitation. Ecology 94, 1687–1696. doi: 10.1890/12-1006.1 24015513

[B9] Currie D. J. Mittelbach G. G. Cornell H. V. Field R. Guégan J.-F. Hawkins B. A.. (2004). Predictions and tests of climate-based hypotheses of broad-scale variation in taxonomic richness. Ecol. letters. 7, 1121–1134. doi: 10.1111/j.1461-0248.2004.00671.x

[B10] Durack P. J. Wijffels S. E. Matear R. J. (2012). Ocean salinities reveal strong global water cycle intensification during 1950 to 2000. Science 336, 455–458. doi: 10.1126/science.1212222 22539717

[B11] Eldridge D. J. Poore A. G. Ruiz-Colmenero M. Letnic M. Soliveres S. (2016). Ecosystem structure, function, and composition in rangelands are negatively affected by livestock grazing. Ecol. Appl. 26, 1273–1283. doi: 10.1890/15-1234 27509764

[B12] Eskelinen A. Virtanen R. (2005). Local and regional processes in low-productive mountain plant communities: The roles of seed and microsite limitation in relation to grazing. Oikos 110, 360–368. doi: 10.1111/j.0030-1299.2005.13579.x

[B13] Fu B. J. Wang S. Liu Y. Liu J. B. Liang W. Miao C. Y. (2017). Hydrogeomorphic ecosystem responses to natural and anthropogenic changes in the loess plateau of China. Annu. Rev. Earth Planetary Sci. 45, 223–243. doi: 10.1146/annurev-earth-063016-020552

[B14] Gao J. Carmel Y. (2020). Can the intermediate disturbance hypothesis explain grazing–diversity relations at a global scale? Oikos 129, 493–502. doi: 10.1111/oik.06338

[B15] Gibson D. J. (2009). “Grasses and grassland ecology,” in Grasses and grassland ecology (Oxford University Press, Oxford, UK).

[B16] Grubb P. J. (1977). The maintenance of species-richness in plant communities: The importance of the regeneration niche. Biol. Rev. 52, 107–145. doi: 10.1111/j.1469-185X.1977.tb01347.x

[B17] Herrero-Jauregui C. Oesterheld M. (2018). Effects of grazing intensity on plant richness and diversity: A meta-analysis. Oikos 127, 757–766. doi: 10.1111/oik.04893

[B18] Huxman T. E. Snyder K. A. Tissue D. Leffler A. J. Ogle K. Pockman W. T.. (2004). Precipitation pulses and carbon fluxes in semiarid and arid ecosystems. Oecologia 141, 254–268. doi: 10.1007/s00442-004-1682-4 15338414

[B19] Kelemen A. Tölgyesi C. Valkó O. Deák B. Miglécz T. Fekete R.. (2019). Density-dependent plant–plant interactions triggered by grazing. Front. Plant Science. 10. doi: 10.3389/fpls.2019.00876 PMC6624794 31333709

[B20] Knapp A. K. Blair J. M. Briggs J. M. Collins S. L. Hartnett D. C. Johnson L. C.. (1999). The keystone role of bison in north american tallgrass prairie - bison increase habitat heterogeneity and alter a broad array of plant, community, and ecosystem processes. Bioscience 49, 39–50. doi: 10.2307/1313492

[B21] Knapp A. K. Fay P. A. Blair J. M. Collins S. L. Smith M. D. Carlisle J. D.. (2002). Rainfall variability, carbon cycling, and plant species diversity in a mesic grassland. Science 298, 2202–2205. doi: 10.1126/science.1076347 12481139

[B22] Koerner S. E. Burkepile D. E. Fynn R. W. Burns C. E. Eby S. Govender N.. (2014). Plant community response to loss of large herbivores differs between north american and South African savanna grasslands. Ecology 95, 808–816. doi: 10.1890/13-1828.1 24933802

[B23] Koerner S. E. Smith M. D. Burkepile D. E. Hanan N. P. Avolio M. L. Collins S. L.. (2018). Change in dominance determines herbivore effects on plant biodiversity. Nat. Ecol. Evol. 2, 1925–1932. doi: 10.1038/s41559-018-0696-y 30374174

[B24] Korell L. Auge H. Chase J. M. Harpole W. S. Knight T. M. (2021). Responses of plant diversity to precipitation change are strongest at local spatial scales and in drylands. Nat. Commun. 12, 2489. doi: 10.1038/s41467-021-22766-0 33941779 PMC8093425

[B25] Lefcheck J. S. (2016). Piecewisesem: Piecewise structural equation modelling in r for ecology, evolution, and systematics. Methods Ecol. Evolution. 7, 573–579. doi: 10.1111/2041-210x.12512

[B26] Li C. Fu B. Wang S. Stringer L. C. Wang Y. Li Z.. (2021). Drivers and impacts of changes in China’s drylands. Nat. Rev. Earth Env. 2, 858–873. doi: 10.1038/s43017-021-00226-z

[B27] Liang M. Gornish E. S. Mariotte P. Chen J. Liang C. (2019). Foliar nutrient content mediates grazing effects on species dominance and plant community biomass. Rangeland Ecol. Management. 72, 899–906. doi: 10.1016/j.rama.2019.08.001

[B28] Liang M. W. Smith N. G. Chen J. Q. Wu Y. T. Guo Z. W. Gornish E. S.. (2021). Shifts in plant composition mediate grazing effects on carbon cycling in grasslands. J. Appl. Ecology. 58, 518–527. doi: 10.1111/1365-2664.13824

[B29] McNaughton S. J. Oesterheld M. Frank D. A. Williams K. J. (1989). Ecosystem-level patterns of primary productivity and herbivory in terrestrial habitats. Nature 341, 142–144. doi: 10.1038/341142a0 2779651

[B30] Miao L. Li S. Zhang F. Chen T. Shan Y. Zhang Y. (2020). Future drought in the dry lands of asia under the 1.5 and 2.0 °c warming scenarios. Earth's Future. 8, e2019EF001337. doi: 10.1029/2019EF001337

[B31] Mortensen B. Danielson B. Harpole W. S. Alberti J. Arnillas C. A. Biederman L.. (2018). Herbivores safeguard plant diversity by reducing variability in dominance. J. Ecology. 106, 101–112. doi: 10.1111/1365-2745.12821

[B32] Olff H. Ritchie M. E. (1998). Effects of herbivores on grassland plant diversity. Trends Ecol. Evolution. 13, 261–265. doi: 10.1016/S0169-5347(98)01364-0 21238294

[B33] Petermann J. S. Buzhdygan O. Y. (2021). Grassland biodiversity. Curr. Biol. 31, R1195–R1201. doi: 10.1016/j.cub.2021.06.060 34637731

[B34] Reich P. B. Tilman D. Isbell F. Mueller K. Hobbie S. E. Flynn D. F.. (2012). Impacts of biodiversity loss escalate through time as redundancy fades. Science 336, 589–592. doi: 10.1126/science.1217909 22556253

[B35] Shi Z. Lin Y. Wilcox K. R. Souza L. Jiang L. F. Jiang J.. (2018). Successional change in species composition alters climate sensitivity of grassland productivity. Global Change Biol. 24, 4993–5003. doi: 10.1111/gcb.14333 29851205

[B36] Sloat L. L. Gerber J. S. Samberg L. H. Smith W. K. Herrero M. Ferreira L. G.. (2018). Increasing importance of precipitation variability on global livestock grazing lands. Nat. Climate Change. 8, 214–218. doi: 10.1038/s41558-018-0081-5

[B37] Smit C. Rietkerk M. Wassen M. J. (2009). Inclusion of biotic stress (consumer pressure) alters predictions from the stress gradient hypothesis. J. Ecology. 97, 1215–1219. doi: 10.1111/j.1365-2745.2009.01555.x

[B38] Smith M. D. Knapp A. K. Collins S. L. (2009). A framework for assessing ecosystem dynamics in response to chronic resource alterations induced by global change. Ecology 90, 3279–3289. doi: 10.1890/08-1815.1 20120798

[B39] Smith M. D. Koerner S. E. Avolio M. L. Komatsu K. J. Eby S. Forrestel E. J.. (2022). Richness, not evenness, varies across water availability gradients in grassy biomes on five continents. Oecologia 199, 649–659. doi: 10.1007/s00442-022-05208-6 35833986

[B40] Stohlgren T. J. Schell L. D. Vanden Heuvel B. (1999). How grazing and soil quality affect native and exotic plant diversity in rocky mountain grasslands. Ecol. Applications. 9, 45–64. doi: 10.1890/1051-0761(1999)009

[B41] Suttle K. B. Thomsen M. A. Power M. E. (2007). Species interactions reverse grassland responses to changing climate. Science 315, 640–642. doi: 10.1126/science.1136401 17272720

[B42] Tilman D. (2000). Causes, consequences and ethics of biodiversity. Nature 405, 208–211. doi: 10.1038/35012217 10821280

[B43] Tóth E. Deák B. Valkó O. Kelemen A. Miglécz T. Tóthmérész B.. (2018). Livestock type is more crucial than grazing intensity: Traditional cattle and sheep grazing in short-grass steppes. Land Degradation Dev. 29, 231–239. doi: 10.1002/ldr.2514

[B44] Trindade D. P. F. Carmona C. P. Partel M. (2020). Temporal lags in observed and dark diversity in the anthropocene. Glob Chang Biol. 26, 3193–3201. doi: 10.1111/gcb.15093 32282128

[B45] Ulrich Sommer Worm B. (2002). “Competition and coexistence,” in Competition and coexistence (Springer, Berlin).

[B46] van Wieren S. E. Bakker J. P. Gordon I. J. Prins H. H. T. (2008). “The impact of browsing and grazing herbivores on biodiversity,” in The ecology of browsing and grazing (Springer Berlin Heidelberg, Berlin, Heidelberg). doi: 10.1007/978-3-540-72422-3_10

[B47] Voigt W. Perner J. Davis A. J. Eggers T. Schumacher J. Bährmann R.. (2003). Trophic levels are differentially sensitive to climate. Ecology 84, 2444–2453. doi: 10.1890/02-0266

[B48] Wang Z. He Y. Niu B. Wu J. Zhang X. Zu J.. (2020). Sensitivity of terrestrial carbon cycle to changes in precipitation regimes. Ecol. Indicators. 113, 106223. doi: 10.1016/j.ecolind.2020.106223

[B49] Worm B. Lotze H. K. Hillebrand H. Sommer U. (2002). Consumer versus resource control of species diversity and ecosystem functioning. Nature 417, 848–851. doi: 10.1038/nature00830 12075351

[B50] Wu Y. Guo Z. Li Z. Liang M. Tang Y. Zhang J.. (2022). The main driver of soil organic carbon differs greatly between topsoil and subsoil in a grazing steppe. Ecol. Evolution. 12, e9182. doi: 10.1002/ece3.9182 PMC9353232 35949532

[B51] Yan H. Li F. Liu G. (2023). Diminishing influence of negative relationship between species richness and evenness on the modeling of grassland α-diversity metrics. Front. Ecol. Evol. 11. doi: 10.3389/fevo.2023.1108739

[B52] Zhou S. Yu B. Zhang Y. (2023). Global concurrent climate extremes exacerbated by anthropogenic climate change. Sci. Advances. 9, eabo1638. doi: 10.1126/sciadv.abo1638 36897946 PMC10005174

