# Supplementary material for: Precipitation variation: a key factor regulating plant diversity in semi-arid livestock grazing lands
Source: Front Plant Sci. 2024 Feb 28;15:1294895. doi: 10.3389/fpls.2024.1294895 (PMC11027165; doi:10.3389/fpls.2024.1294895)
Supplement: Supplementary file 1 [file DataSheet_1.docx]

Supplementary Material

# Supplementary Figures and Tables

**Supplementary Figure**


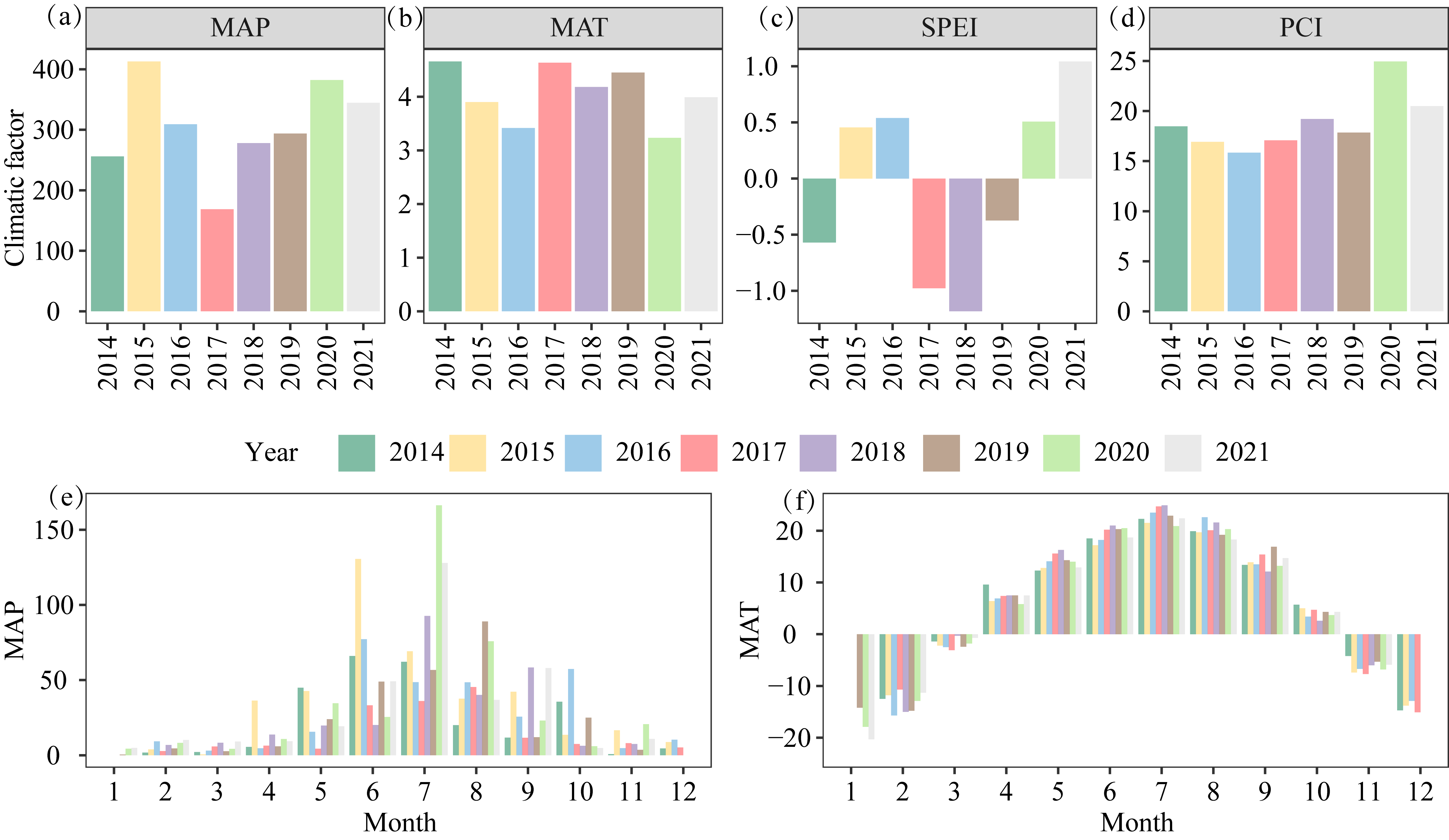


**Figure S1.** The inter-annual and intra-annual distribution of climate factors. Panel (a) illustrates the inter-annual mean annual precipitation (MAP) from 2014 to 2021. Panel (b) shows the inter-annual mean annual temperature (MAT) from 2014 to 2021. Panel (c) displays the inter-annual standardized precipitation evapotranspiration index (SPEI) from 2014 to 2021. Panel (d) depicts the inter-annual precipitation concentration index (PCI) from 2014 to 2021. Panel (e) presents the mean annual precipitation for each month across different years. Panel (f) demonstrates the mean annual temperature for each month across different years from 2014 to 2021.


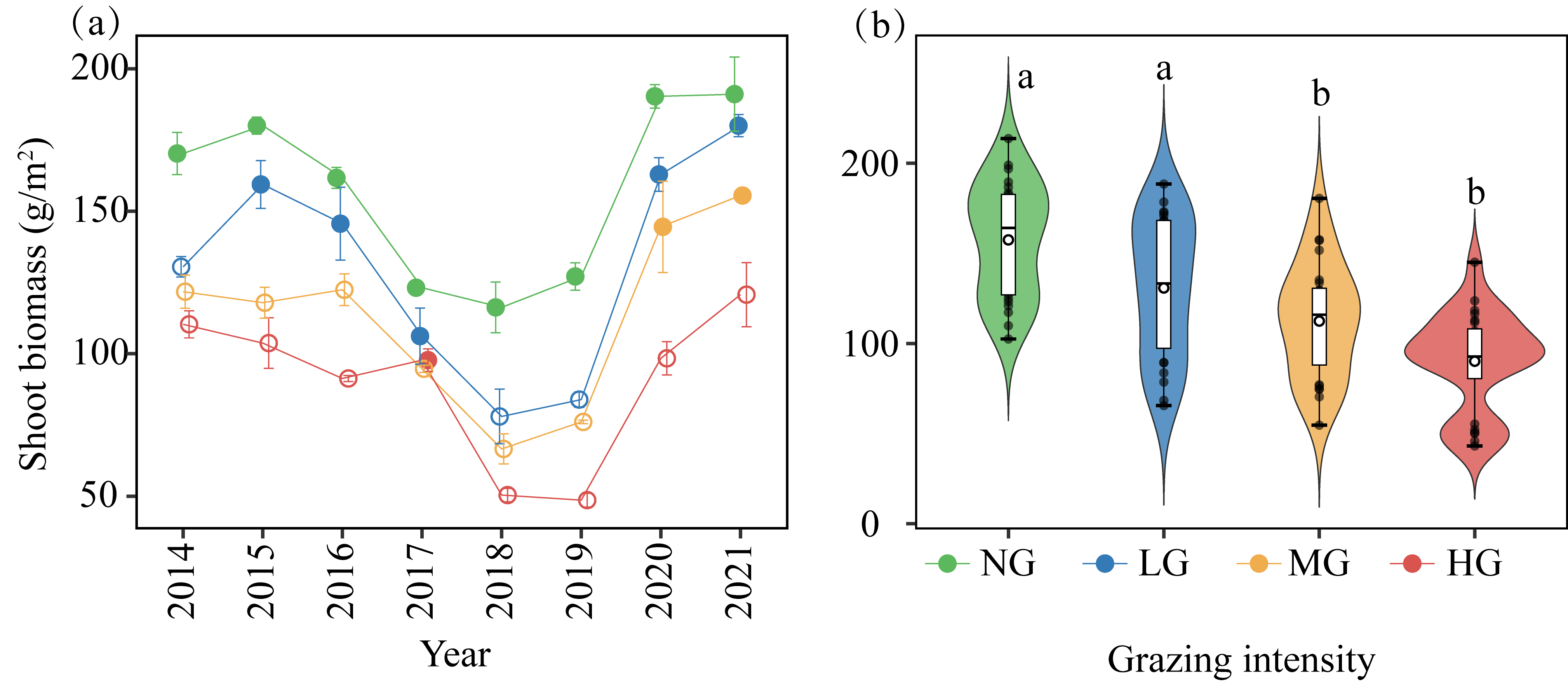


**Figure S2.** The impacts of grazing intensity and year on plant shoot biomass. Panel (a) shows the inter-annual variation in shoot biomass under different grazing intensities. Panel (b) presents the differences in shoot biomass among different grazing intensities. The different lowercase letters in the figure indicate significant differences, with a significance level of p<0.05. Key: NG = no grazing (green), LG = low grazing intensity (blue), MG = medium grazing intensity (yellow), HG = high grazing intensity (red).


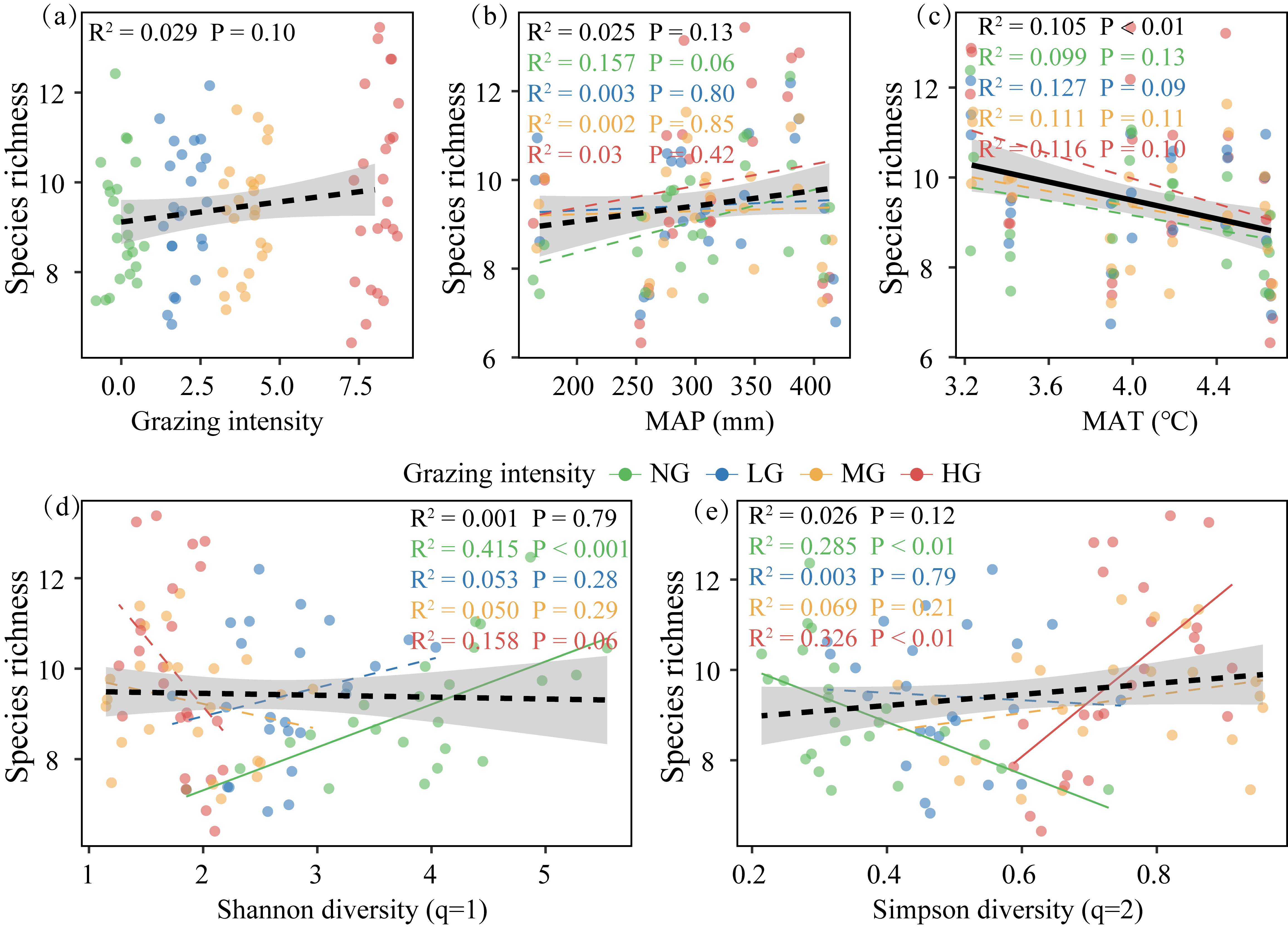


**Figure S3.** The impacts of grazing and climate on plant communities: (a) Relationship between grazing intensity and species richness. (b) Relationship between mean annual precipitation (MAP) and species richness. (c) Relationship between mean annual temperature (MAT) and species richness. (d) Relationship between Shannon diversity (Shannon diversity) and species richness. (e) Relationship between Simpson dominance index (Simpson diversity) and species richness. Solid lines indicate a significant correlation, while dashed lines indicate no significant relationship, with a significance level of p<0.05. Key: NG = no grazing (green), LG = low grazing intensity (blue), MG = medium grazing intensity (yellow), HG = high grazing intensity (red).


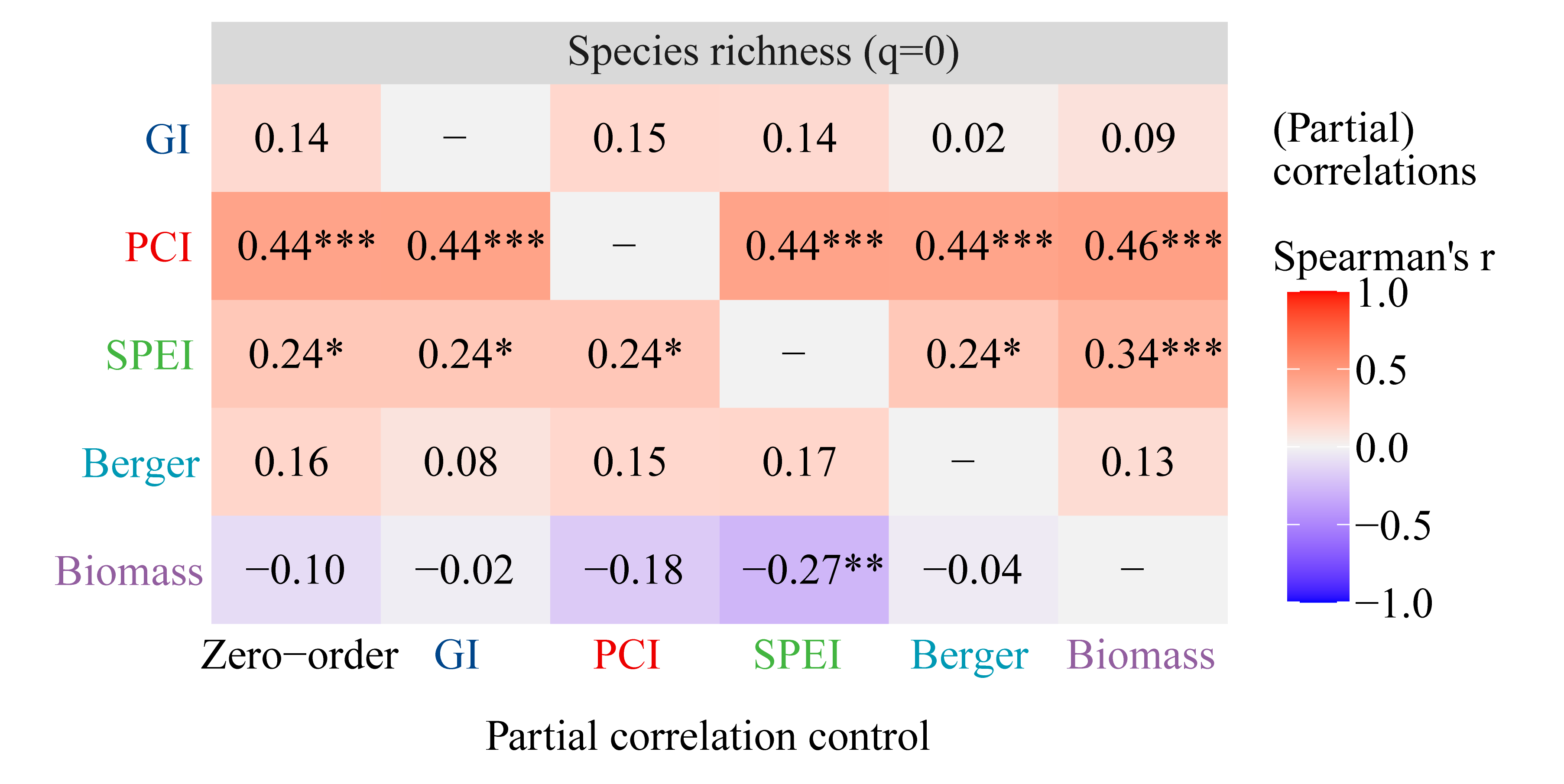


**Figure S4.** The partial correlation (Spearman's r) between species richness and grazing intensity (GI), climate factors (PCI and SPEI), and plant community properties (Berger-parker and Shoot biomass). The color and number indicate the correlation coefficient, with red representing a positive correlation and blue representing a negative correlation. The significance levels are as follows: P＜0.10; *: P＜0.05; **P＜0.01; and ***: P＜0.001. Key: PCI = precipitation concentration index, SPEI = standardized precipitation evapotranspiration index, Berger = Berger-parker dominance index, Biomass = Shoot biomass.


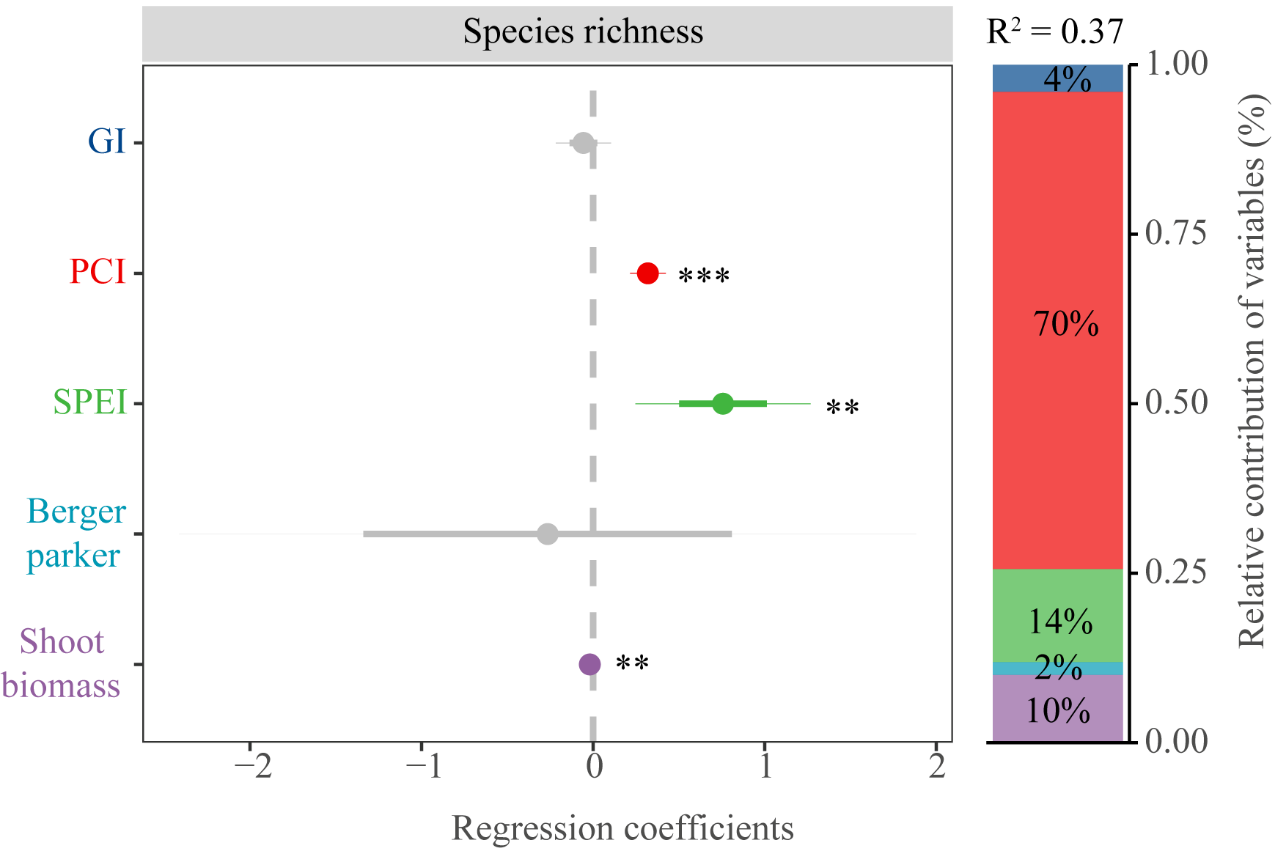


**Figure S5** Herbivory (GI), climate (PCI and SPEI) and plant community (Berger-Parker and Shoot biomass) effects on species richness. Shown are the regression coefficients from the multiple regression model, quantifying the primary factors driving species richness. Significant level: *p < 0.05, **p < 0.01, ***p < 0.001. Key: GI= grazing intensity (blue), PCI = precipitation concentration index (red), SPEI = standardized precipitation evapotranspiration index (green), Berger-Parker = dominance index (light blue).

**Supplementary Tables**

**Table S1.** The effects of grazing intensity on biodiversity (i.e., species richness, Shannon diversity, and Simpson diversity) for each year were analyzed (mean ± SE, n=3). Multiple comparisons among grazing intensities were conducted using the Tukey's test. Significant differences between any two groups with different lowercase letters were observed at p < 0.05. Key: NG = no grazing, LG = low grazing intensity, MG = medium grazing intensity, HG = high grazing intensity.

| Year | GI | Species richness | Berger-parker | Simpson | Shannon |
| --- | --- | --- | --- | --- | --- |
|  |  | Mean±se | Mean±se | Mean±se | Mean±se |
| 2014 | NG | 8.33±0.45a | 0.72±0.07a | 0.56±0.08a | 2.68±0.39a |
|  | LG | 7.27±0.12a | 0.69±0.04a | 0.54±0.04a | 2.4±0.15a |
|  | MG | 8.07±0.49a | 0.75±0.04a | 0.61±0.04a | 2.21±0.11a |
|  | HG | 6.93±0.31a | 0.79±0.01a | 0.64±0.01a | 2.07±0.02a |
| 2015 | NG | 8.6±0.4a | 0.57±0.08a | 0.42±0.07a | 3.24±0.41a |
|  | LG | 8±0.66a | 0.66±0.09a | 0.55±0.09a | 2.36±0.28a |
|  | MG | 7.93±0.35a | 0.77±0.07a | 0.64±0.09a | 2.06±0.25a |
|  | HG | 7.6±0.1a | 0.8±0.02a | 0.65±0.03a | 1.95±0.09a |
| 2016 | NG | 8.13±0.35b | 0.51±0.05b | 0.36±0.03b | 3.51±0.27a |
|  | LG | 9.07±0.21ab | 0.63±0.07ab | 0.48±0.05ab | 2.68±0.27ab |
|  | MG | 9.87±0.12a | 0.76±0.09ab | 0.63±0.11ab | 2.17±0.38ab |
|  | HG | 9.2±0.17ab | 0.85±0.01a | 0.73±0.01a | 1.78±0.05b |
| 2017 | NG | 7.93±0.31a | 0.53±0.06b | 0.37±0.05b | 3.64±0.31a |
|  | LG | 9.87±0.6a | 0.59±0.05b | 0.44±0.04b | 2.89±0.28ab |
|  | MG | 9.27±0.4a | 0.81±0.08ab | 0.71±0.11ab | 1.92±0.35bc |
|  | HG | 9.67±0.29a | 0.93±0.02a | 0.87±0.04a | 1.4±0.11c |
| 2018 | NG | 9.73±0.06a | 0.42±0.04b | 0.26±0.02b | 4.8±0.29a |
|  | LG | 9.93±0.49a | 0.54±0.07b | 0.38±0.05b | 3.52±0.35a |
|  | MG | 8.73±0.7a | 0.85±0.05a | 0.74±0.09a | 1.84±0.3b |
|  | HG | 9.6±0.61a | 0.84±0.03a | 0.7±0.05a | 1.9±0.1b |
| 2019 | NG | 8.93±0.5a | 0.48±0.02c | 0.31±0.01c | 4.15±0.13a |
|  | LG | 10±0.44a | 0.69±0.03b | 0.51±0.04b | 2.59±0.13b |
|  | MG | 10.6±0.62a | 0.92±0.03a | 0.85±0.05a | 1.48±0.16c |
|  | HG | 11.53±0.74a | 0.93±0a | 0.87±0a | 1.44±0.01c |
| 2020 | NG | 10.4±1a | 0.42±0.05c | 0.28±0.03c | 4.72±0.45a |
|  | LG | 11.53±0.31a | 0.73±0.03b | 0.55±0.05b | 2.53±0.15b |
|  | MG | 10.8±0.44a | 0.93±0.02a | 0.87±0.04a | 1.44±0.12b |
|  | HG | 12.47±0.29a | 0.86±0.01ab | 0.74±0.02a | 1.89±0.07b |
| 2021 | NG | 10.8±0.17ab | 0.42±0.02b | 0.28±0b | 4.4±0.01a |
|  | LG | 9.73±0.6ab | 0.61±0.02b | 0.43±0.02b | 3.07±0.1b |
|  | MG | 9.13±0.51b | 0.86±0.07a | 0.76±0.11a | 1.77±0.33c |
|  | HG | 12.13±0.65a | 0.89±0.02a | 0.8±0.04a | 1.67±0.14c |
